# Osteogenic Effects of the *Diospyros lotus* L. Leaf Extract on MC3T3-E1 Pre-Osteoblasts and Ovariectomized Mice via BMP2/4 and TGF β Pathways

**DOI:** 10.3390/nu16081247

**Published:** 2024-04-22

**Authors:** Soyeon Hong, Nadzeya Lazerka, Byeong Jun Jeon, Jeong Do Kim, Saruul Erdenebileg, Chu Won Nho, Gyhye Yoo

**Affiliations:** 1 Smart Farm Research Center, Gangneung Institute of Natural Products, Korea Institute of Science and Technology (KIST), Gangneung 25451, Republic of Korea; hongsso@kist.re.kr (S.H.); nadzeya.lazerka@kist.re.kr (N.L.); jeonbyeongjun@kist.re.kr (B.J.J.); kimjeongdo@kist.re.kr (J.D.K.); 619008@kist.re.kr (S.E.); cwnho@kist.re.kr (C.W.N.); 2 Division of Natural Product Applied Science, KIST School, University of Science and Technology (UST), Seoul 02792, Republic of Korea

**Keywords:** osteoporosis, *Diospyros lotus* leaf extract (DLE), osteoblast differentiation, bone morphogenic protein 2/4 (BMP2/4), transforming growth factor β (TGF β)

## Abstract

Osteoporosis, a disease defined by the primary bone strength due to a low bone mineral density, is a bone disorder associated with increased mortality in the older adult population. Osteoporosis is mainly treated via hormone replacement therapy, bisphosphates, and anti-bone resorption agents. However, these agents exert severe side effects, necessitating the development of novel therapeutic agents. Many studies are focusing on osteogenic agents as they increase the bone density, which is essential for osteoporosis treatment. Here, we aimed to investigate the effects of *Diospyros lotus* L. leaf extract (DLE) and its components on osteoporosis in MC3T3-E1 pre-osteoblasts and ovariectomized mice and to elucidate the underlying related pathways. DLE enhanced the differentiation of MC3T3-E1 pre-osteoblasts, with a 1.5-fold elevation in ALP activity, and increased the levels of osteogenic molecules, RUNX family transcription factor 2, and osterix. This alteration resulted from the activation of bone morphogenic protein 2/4 (BMP2/4) and transformation of growth factor β (TGF β) pathways. In ovariectomized mice, DLE suppressed the decrease in bone mineral density by 50% and improved the expression of other bone markers, which was confirmed by the 3~40-fold increase in osteogenic proteins and mRNA expression levels in bone marrow cells. The three major compounds identified in DLE exhibited osteogenic and estrogenic activities with their aglycones, as previously reported. Among the major compounds, myricitrin alone was not as strong as whole DLE with all its constituents. The osteogenic activity of DLE was partially suppressed by the inhibitor of estrogen signaling, indicating that the estrogenic activity of DLE participated in its osteogenic activity. Overall, DLE suppresses osteoporosis by inducing osteoblast differentiation.

## 1. Introduction

Osteoporosis is a metabolic skeletal disorder characterized by a low bone mass and strength and an increased risk of bone microarchitecture destruction [1,2]. According to the World Health Organization, over 10 million people in the United States and 30 million people in Europe are annually diagnosed with osteoporosis [3]. Among the known causes of osteoporosis, the menopause is a critical factor, inducing rapid bone loss in women, as female sex hormones are essential regulators of bone remodeling and related cells. Due to the menopause, osteoporosis is generally observed in one in every two women and one in every five men aged >50 years. Therefore, women undergoing menopause are treated via hormone replacement therapy (HRT), which suppresses osteoporosis. However, HRT is not a permanent cure and is associated with a risk of breast cancer when used over the long term [4,5].

Bone is an active tissue undergoing constant cell resorption and formation, which is known as bone remodeling [6]. Two major cell types are involved in bone remodeling: osteoblasts and osteoclasts. Osteoclasts are bone resorptive cells, whereas osteoblasts are bone-forming cells [7,8]. Under normal conditions, a balance between bone formation and resorption maintains homeostasis of an appropriate bone density and mass. However, any imbalance between bone resorption and formation due to various factors, such as the menopause, induces osteoporosis. The currently available treatments for osteoporosis focus on the inactivation of osteoclasts using bisphosphates and calcitonin [9]. However, novel therapeutic agents are needed to activate osteoblasts as the current agents cause severe side effects, and the suppression of bone resorption is not a fundamental cure for osteoporosis [10].

Globally, the importance of finding treatments derived from natural products that have fewer side effects and better therapeutic effects has been highlighted. *Diospyros lotus* L. (DL) is a date plum generally found in south and southeast Europe. This plant exerts anti-inflammatory, anti-diabetic, and anti-tumor effects [11,12,13,14,15]. DL extract contains several phytochemicals, including gallic acid, kaempferol, myricetin, and their derivatives. Myricetin is a representative phytoestrogen with osteogenic activity, whereas kaempferol is a flavonoid found in several medicinal plants that promotes bone formation in vitro and in vivo [16,17]. However, whether DL exerts beneficial effects on bone health has not yet been studied. The objective of this study is to demonstrate the effect of DL on bone and bone cells. Therefore, in this study, we evaluated whether DL extract suppresses osteoporosis by mediating the osteogenic activity *in vitro* and *in vivo*.

## 2. Materials and Methods

### 2.1. Plant Materials

DL was purchased from Herbmaul (Cheongju, South Korea). Dried plants (50 g) were extracted with 50% ethanol for three days (72 h) at room temperature [15]. The extract and supernatants were filtered through a filter paper, and the filtrate was evaporated using the SpeedVac Concentrator (SPD2010; Thermo Scientific, Waltham, MA, USA) until only solid remained.

### 2.2. High-Performance Liquid Chromatography (HPLC) and Quadrupole Time-of-Flight (Q-TOF) Mass Spectrometry Analyses of Phytochemicals in DL

Next, the 50% ethanol extract of *D. lotus* (DLE) was analyzed using an HPLC system (Agilent 1260 Infinity; Waldbronn, Germany) equipped with a YMC-Triart C18 column (150 × 4.6 mm, S-5 μm, 12 nm) via linear gradient elution with 0.1% aqueous formic acid (solvent A) and acetonitrile (solvent B) [14]. HPLC analysis was conducted at a flow rate of 1 mL min^−1^ using a linear gradient elution of 5–10% solvent B in solvent A for 10 min, 10–40% solvent B in solvent A for 40 min, and 40–100% solvent B in solvent A for 4 min. Each peak was monitored at 280 nm. The sample injection volume was 10 μL (10 mg mL^−1^) at a 30 °C column temperature.

The mass spectrum of DLE was obtained using an Agilent 1290 Infinity UHPLC system equipped with an Agilent RRHD Eclipse plus C18 column (2.1 × 50 mm, 1.8 μm) and a Q-TOF mass spectrometer (Agilent G6550A) at the Korea Basic Science Institute (KBSI). Solvent A was water containing 0.1% formic acid, and solvent B was acetonitrile containing 0.1% formic acid. HPLC analysis was conducted at a flow rate of 0.3 mL min^−1^ using a linear gradient elution of 5% B (3 min), 5–50% B (27 min), and 50–80% B (10 min) at 280 nm. The sample injection volume for LC/MS was 1 μL (10 mg mL^−1^) at a 30 °C column temperature. MS data were acquired within the mass range of *m*/*z* 80–1700 at a scan rate of 1 spectra/s.

### 2.3. Cell Culture

Cell cultures were conducted as previously described [18]. MC3T3-E1 murine osteoblastic cells and MCF-7 human breast cancer cells were purchased from the American Type Culture Collection (Manassas, VA, USA). MC3T3-E1 cells were seeded in T75 flasks, maintained at 37 °C with 5% CO_2_, and cultured in the alpha-minimum essential medium (Gibco, Baithersburg, MD, USA) containing 1% penicillin/streptomycin (Gibco) and 10% fetal bovine serum (Gibco). For osteoblast differentiation, MC3T3-E1 cells were differentiated using osteoblastic differentiation medium (DM), which was prepared by adding 10 mM β-glycerophosphate and 50 μg mL^−1^ L-ascorbic acid (Sigma-Aldrich, St. Louis, MO, USA) to the culture medium. Cells treated with DM for six days were used for ALP activity, protein, and mRNA analyses. MCF-7 cells were cultured in MEM containing 10% fetal bovine serum and 1% penicillin streptomycin in a 5% CO_2_ incubator.

### 2.4. ALP Activity

MC3T3-E1 cells were seeded at 5 × 10^4^ cells mL^−1^ in a 24-well plate and treated with 25 and 50 μg mL^−1^ DLE in DM for six days to assess the ALP activity. To evaluate the effect of estrogen signaling on osteoblast differentiation, 10 μM ICI182,780 (Sigma-Aldrich), the inhibitor of estrogen signaling, was co-treated with or without DLE. Finally, the absorbance was measured at 405 nm using a multi-detection microplate reader (Synergy HT; BioTek Instruments, Winooski, VT, USA). All experiments using cells were performed in three biological replicates and three technical replicates.

### 2.5. Luciferase Reporter Assays

For phytoestrogen stimulation, MCF-7 cells were cultured in the experimental medium, which contained phenol-red-free MEM supplemented with 10% charcoal dextran-treated fetal bovine serum, 2 mmol L^−1^ L-glutamine, 1 mmol L^−1^ sodium pyruvate, and 1% penicillin streptomycin in a 5% CO_2_ incubator. MCF7 cells were transfected with an estrogen response element luciferase plasmid and qRL-CMV plasmid using iN-fect *in vitro* transfection reagent (INtRON, Seoul, South Korea) according to the manufacturer’s instructions. All cells were allowed to reach 50–80% confluence prior to transfection. After 24 h, the medium was replaced with fresh medium containing DLE (25–50 μg mL^−1^) for 24 h. Then, cells were lysed and assayed for luciferase expression using a Dual Luciferase Assay kit (Promega, Madison, WI, USA) according to the manufacturer’s instructions. Luciferase activity was detected using a Synergy HT multi-microplate reader (BioTek Instruments, Winooski, VT, USA). Renilla luciferase activity was used to normalize the transfection efficiencies.

### 2.6. Western Blotting

Western blots were conducted as previously described [18]. Total protein was extracted using the radioimmunoprecipitation assay buffer. The extract was centrifuged (14,000 rpm) at 4 °C for 30 min. The supernatant was collected, and protein levels were quantified using the Bradford assay. Proteins were separated via 10% sodium dodecyl sulfate-polyacrylamide gel electrophoresis and transferred onto a nitrocellulose membrane for 2 h. Subsequently, the membrane was blocked with 3% BSA in phosphate-buffered saline containing 0.1% Tween 20 (PBST) for 2 h at room temperature and incubated with primary antibodies at 4 °C overnight. After washing thrice with PBST, the membrane was incubated with secondary antibodies at 37 °C for 4 h. Antibodies against β-catenin, p-Smad1/5, and p-Smad2/3 and secondary anti-rabbit antibodies were purchased from Cell Signaling Technology (Danvers, MA, USA), and BMP2/4, TGFβ, RUNX2, OSX, OPN, Col1a1, β-actin and secondary anti-mouse antibodies were purchased from Santa Cruz Biotechnology (Dallas, TX, USA). Bands were detected using a Pierce^®^ ECL Western Blotting Substrate (Thermo Fisher Scientific, Waltham, MA, USA). The resulting bands were normalized using β-actin as a loading control.

### 2.7. Quantitative Reverse Transcription-Polymerase Chain Reaction (qRT-PCR)

qRT-PCRs were conducted as previously described [18]. Total RNA was extracted using a total RNA isolation kit (GeneALL Biotechnology, Seoul, South Korea) according to the manufacturer’s instructions. Then, total RNA was quantified using a NanoDrop spectrophotometer. cDNA was synthesized using a PrimeScript 1st Strand cDNA Synthesis Kit (TaKaRa, Osaka, Japan). qRT-PCR analysis was performed using SYBR Green Master Mix (Roche, Switzerland) and a Light Cycler 480 Real-Time PCR System (Roche, Switzerland). The PCR conditions were set as follows: 94 °C for 1 min (denaturation), 55–58 °C for 30 s (annealing), and 72 °C for 1 min (extension). All primers used in this study are listed in Table 1. The resulting products were normalized using β-actin.

### 2.8. Animal Surgery and Treatment

All Animal Care and Experimental Protocols and all animal experiments were reviewed and approved by the Korea Institute of Science and Technology (KIST-2022-153-1).

Animal experiments were conducted as previously described [18]. To construct an osteoporosis-inducing animal test model, C57BL/6J (7-week-old, female) mice were purchased from Central Lab Animal, Inc. (Seoul, South Korea). Mouse were kept under a 12 h:12 h light/dark photoperiod at a constant temperature (25 ± 2 °C), and food and water were available *ad libitum*. To reduce errors due to mice stress, acclimatization was performed for 1 week in an approved facility. Ovariectomy (OVX) was performed by removing both ovaries, and sham surgery was performed without removing the ovaries under the same respiratory anesthesia conditions. The experimental diet was the American Institute of Nutrition (AIN)-93M diet (Dyets Inc., Bethlehem, PA, USA). All samples were prepared in a 0.5% carboxymethyl cellulose (CMC) solution containing 0.5% sesame oil and 0.5% dimethyl sulfoxide to dissolve them properly [16]. Except for the SHAM group (*n* = 7), OVX mice were randomly divided into the following five groups: OVX mice treated with a 0.5% carboxymethyl cellulose (CMC) solution containing 0.5% sesame oil and 0.5% dimethyl sulfoxide (negative control; OVX, *n* = 6), OVX mice treated with 0.1 mg kg^−1^ β-estradiol (Sigma-Aldrich, St. Louis, MO, USA) and 1 mg kg^−1^ progesterone (Sigma-Aldrich, St. Louis, MO, USA) dissolved in 0.5% CMC solution containing 0.5% sesame oil and 0.5% DMSO (positive control, E + P, *n* = 6), and OVX mice treated with 20 and 100 mg kg^−1^ DLE 0.5% CMC solution containing 0.5% sesame oil and 0.5% DMSO (DLE 20 and 100 mg kg^−1^, *n* = 5~7). After two weeks of recovery from the OVX, administration of all samples was started in the mice orally and continued for 12 weeks. All mice care was in accordance with ethical standards and body weight was measured weekly. Mice were sacrificed via cervical dislocation under anesthesia, blood was collected for serum isolation, and the uterus and femur were collected for analysis. All left femurs were fixed with formalin for micro-computed tomography (micro-CT) and hematoxylin and eosin and immunohistochemical staining, and all right femurs were used to isolate the bone marrow cells.

### 2.9. Micro-CT

Micro-CT analysis of the distal femoral trabecular bone 14 weeks after OVX surgery was performed as previously described [18,19]. Briefly, the collected femurs were stored in formalin and scanned using SkyScan 1172 (BRUKER Micro-CT Corp., Kontich, Belgium). The original image was reconstructed using NRecon software v1.7.3.2 (BRUKER micro-CT Corp). To analyze the reconstructed images to determine micro-CT indices, CTan integrated software v.1.17.7.2 (BRUKER micro-CT Corp) was used to analyze the bone mineral density (BMD), bone surface area/bone volume (BS/BV), bone surface area/total volume (BS/TV), trabecular plate number (Tb. N), bone volume/total volume (BV/TV), trabecular spacing (Tb.Sp), trabecular thickness (Tb.Th), and cortical bone thickness (Ct.Th).

### 2.10. Determination of Serum Osteocalcin (OCN) Levels

Serum analyses were conducted as previously described [18]. Plasma samples were analyzed. The plasma sample was prepared by centrifuging blood obtained from cardiac blood collection at 14,000 rpm at 4 °C for 30 min and separating the supernatant. Supernatant separation was performed on ice, and all samples were stored at –80 °C until use. Based on the pre-test results, plasma samples were used without dilution. Serum OCN levels were measured using a Gla-OC EIA Kit (TaKaRa). All tests were performed according to the manufacturers’ instructions.

### 2.11. Statistical Analyses

For all analyses, data are represented as the means ± standard error of the mean. Statistical analyses were conducted via a one-way ANOVA, followed by Duncan’s multiple range test as a post hoc test using the software package Graph-Pad Prism version 10.2.1 (GraphPad, La Jolla, CA, USA). The critical level of significance was set at *p* < 0.05.

## 3. Results and Discussions

### 3.1. DLE Enhances Osteoblast Differentiation by Inducing Osteogenic Signaling

**Figure 1:**
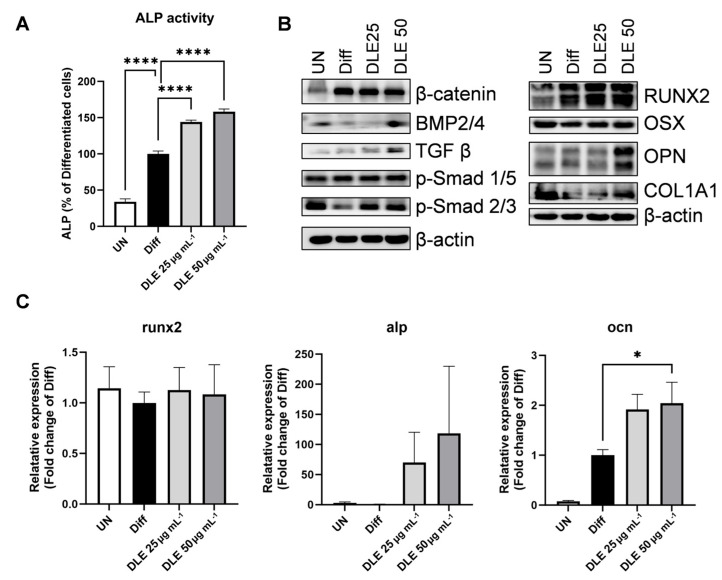
Osteogenic activity of DLE on MC3T3E1 pre-osteoblast cells markers. MC3T3E1 cells were differentiated in DM media with/without 25 or 50 μg mL^−1^ DLE for 6 days. (**A**) ALP activity, (**B**) protein expression via Western blot, and (**C**) mRNA expression of osteogenic markers via qRT-PCR. (* *p* < 0.05; **** *p* < 0.0001).

To investigate the effect of DLE on osteoblast differentiation, the ALP activity was estimated in MC3T3-E1 pre-osteoblasts differentiated with β-glycerolphosphate and L-ascorbic acid with or without DLE for six days. DLE increased the ALP activity in a dose-dependent manner compared with that in differentiated cells (Figure 1A, *p <* 0.0001). Evaluation of the osteogenic markers at the protein and mRNA levels revealed alterations in the critical regulators of osteogenic signaling. DLE treatment increased the protein expression levels of RUNX family transcription factor 2 (RUNX2), a major transcription factor involved in osteogenic signaling, and collagen type I alpha 1 (COL1A1), an essential component of the extracellular matrix for bone formation (Figure 1B). Levels of bone morphogenetic protein (BMP)-2/4 and transforming growth factor (TGF)-β, initial modulators of osteoblast differentiation, were elevated by DLE. Additionally, DLE increased the mRNA expression levels of ocn (*p <* 0.05), a calcium-binding protein that concentrates calcium in the bone, and alp, a representative bone formation marker (Figure 1C).

Development of novel therapeutic agents for osteoblast differentiation and bone formation is necessary to recover bone density, because osteoporosis is a result of less bone formation and more bone resorption [6,9]. Of course, slowing down bone resorption is one way to treat osteoporosis. However, considering that patients diagnosed with osteoporosis have a low bone density, suppression of bone resorption is not the best option for them. Instead, an increase in bone formation via osteoblast activation could result in enough bone density and fundamental recovery of bone status. Here, we studied the effect of DLE on osteoblast differentiation. DLE modulated BMP2/4 and TGF β, which are known to play an essential role in fetal development through the Smad cascade [6]. These are early inducers of the differentiation of osteoblasts from mesenchymal stem cells via controlling RUNX2 and OSX, indispensable transcription factors for osteoblast differentiation. The effect of DLE on BMP2/4 and TGF β indicates that DLE could be a novel candidate for osteoporosis via induction of osteoblast differentiation, and the effect of DLE is mediated by BMP2/4 and TGF β pathways.

### 3.2. DLE Contains Myricetrin, Quercetrin, and Kaempferol-7-O-Rhamnoside

**Figure 2:**
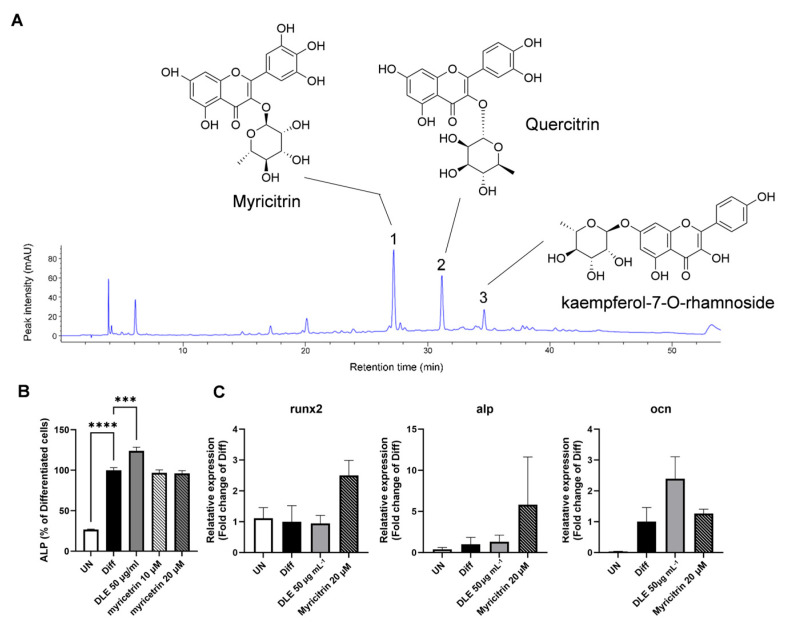
Chemical profiling of DLE and the osteogenic activity of DLE’s components. (**A**) HPLC chromatogram of DLE, (**B**) ALP activity, and (**C**) mRNA expression of osteogenic markers by myricitrin, DLE’s component. (*** *p* < 0.001; **** *p* < 0.0001).

HPLC analysis of DLE revealed three major compounds (Figure 2A). Main peaks 1, 2, and 3 were monitored at retention time points of 27.2, 31.1, and 34.6 min, respectively (Figure 2A). The mass spectra of peaks 1, 2, and 3 exhibited major [M–H]^−^ ions at *m*/*z* 463.0940, 447.0993, and 431.1034, respectively. Finally, peaks 1, 2, and 3 were identified as myricitrin (C_21_H_2_0O_12_), quercitrin (C_21_H_2_0O_11_), and kaempferol-7-*O*-rhamnoside (C_21_H_2_0O_10_), respectively, using Q-TOF mass spectrometry data. We compared the myricitrin authentic standard and peak 1 of DLE to confirm that the retention time and mass were consistent and determined the concentration of myricitrin in DLE. The content of myricitrin in DLE was 122 mg g^−1^ (0.122 mg mL^−1^). This HPLC result indicates that DLE contains significant amounts of flavonoids such as myricitrin, quercitrin, and kaempferol-7-*O*-rhamnoside.

To investigate the effects of the identified compounds on osteoblast differentiation, ALP activity was estimated in MC3T3-E1 pre-osteoblasts differentiated with DLE and myricitrin for six days. Compared with DLE, myricitrin did not increase the ALP activity (Figure 2B). Moreover, compared with DLE, myricitrin increased the mRNA levels of *runx2* and *alp*, but the difference was not significant (Figure 2C).

Consistent with previous reports [14,20], myricitrin, quercitrin, and kaempferol-7-*O*-rhamnoside were identified as the major compounds in DLE. Myricitrin is a myricetin glycoside, and myricetin exhibits estrogenic activity and suppresses osteoporosis via increased osteoblast differentiation and decreased osteoclast activation [21,22]. Quercitrin, a glycoside of quercitrin, exerts protective effects on osteoporosis at 50 mg kg^−1^ [23]. Kaempferol-7-*O*-rhamnoside is a derivative of kaempferol, which possesses anti-oxidant and anti-inflammatory properties [18]. Here, DLE increased osteogenic signaling and suppressed osteoporosis through the activities of these compounds. However, the effects of whole DLE were much stronger than those of the constituent compounds. According to previous reports, 50 mg kg^−1^ of quercitrin has been used to elevated BMD and other bone parameters, while 40 mg kg^−1^ of DLE was utilized in this study. We observed that 40 mg of DLE contained ≤100 µg quercitrin. However, this concentration was insufficient to induce osteogenic activity. Furthermore, 50 µg mL^−1^ of DLE elevated the ALP activity better than 20 µM of myricitrin after six days of differentiation. Therefore, the osteogenic activity of DLE could be due to the synergistic effects of all compounds in DLE. Still, further studies are needed to reveal other potential effects of DLE, like a novel minor compound with osteogenic activity or systemic modification of DLE components during absorption in the gut.

### 3.3. Estrogenic Activity of DLE Partially Mediates the Osteogenic Activity

**Figure 3:**
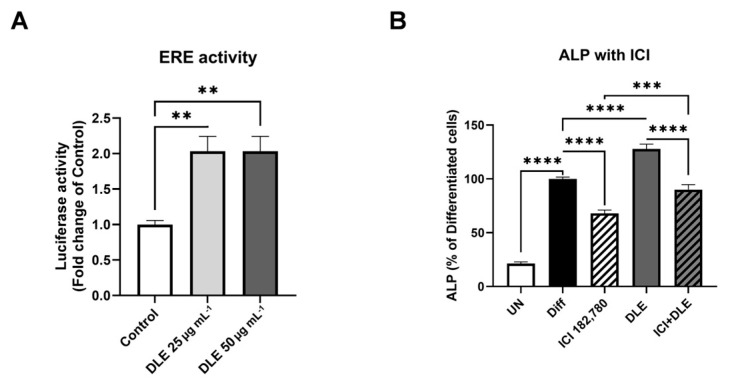
Estrogenic activity of DLE. (**A**) Luciferase activity caused by DLE in MCF7 cells transfected with ERE-luc plasmids, and (**B**) ALP activity modulated by the combination of DLE and/or ICI182780, the inhibitor of estrogen signaling (** *p* < 0.01, *** *p* < 0.001; **** *p* < 0.0001).

As the major compounds identified in DLE were phytoestrogens and as estrogen signaling plays a critical role in bone remodeling, the estrogenic activity of DLE was estimated. Analysis of luciferase activity controlled by ERE revealed that DLE significantly increased the luciferase activity (Figure 3A, *p <* 0.01). To further determine the role of the estrogenic activity of DLE in osteoblast differentiation, ALP activity was estimated in the presence or absence of ICI182,780, the inhibitor of estrogen signaling. ALP activity was elevated by the differentiation medium and was decreased by ICI182,780, and co-treatment with DLE and ICI182,780 affected the ALP activity similar to the effects observed in differentiated cells (Figure 3B, *p <* 0.0001). The level of ALP activity in co-treatment with DLE and ICI182,780 was much higher than that after ICI182,780 treatment (*p <* 0.001).

Estrogenic activities of the major compounds in DLE have been reported in previous studies. Myricetin, quercetin, and kaempferol exhibit strong estrogenic activities and act as phytoestrogens [14]. These findings imply that the estrogenic activity of DLE mediates its osteogenic activity in the bone. However, the combination of DLE and ICI182,780 exhibited a weaker inhibitory effect on ALP than ICI182,780 alone in this study. These data indicate that the osteogenic effects of DLE did not partially result from the estrogenic activity of phytoestrogens. DLE may modulate osteogenic markers independent of estrogenic signaling. Both BMP2/4 and TGFβ are upregulated by DLE and are molecules related to the immune system. Myricetin, quercetin, and kaempferol exert anti-inflammatory and modulatory effects on various types of immune cells [24,25]. Bone marrow has various immune cells, and T cells are a source of BMP2/4 and TGFβ. Therefore, immunomodulation by DLE and its compounds increases the levels of osteogenic inducers BMP2/4 and TGFβ, leading to osteoblast differentiation [26,27,28]. The immunomodulatory effects of DLE may indirectly originate from estrogenic activation. Estrogen signaling suppresses pathogens in the gut and intestinal epithelial barrier permeability, thereby enhancing the immune status [29]. Sufficient estrogen increases the number of regulatory T cells, which secrete TGFβ, and decreases the number of T helper 17 cells, which produce the osteoclast inducers TNFα and the receptor activator of nuclear factor-kappa B ligand [30,31]. Therefore, the effects of DLE may be derived from the recovery of the immune system and gut microbiome via estrogenic activation in the gut. To clarify the mechanism of DLE, further study is required to demonstrate the impact of estrogen signaling and/or the immune system on DLE’s effects.

### 3.4. DLE Suppresses Osteoporosis in OVX Mice

**Figure 4:**
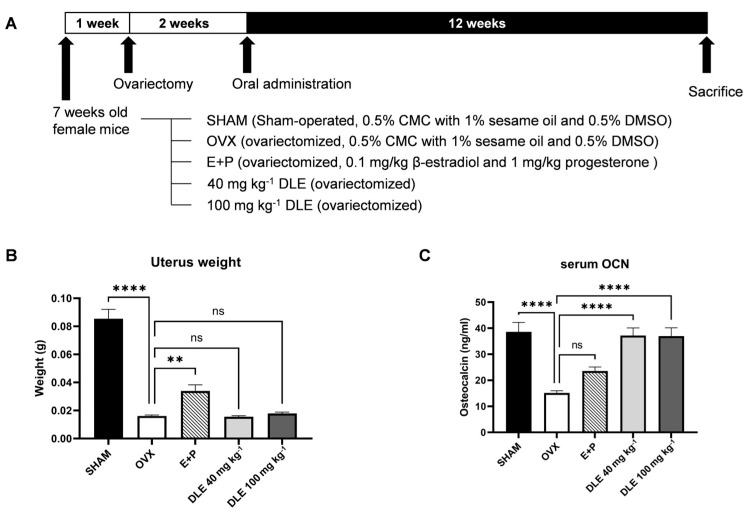
Effects of DLE on physiological markers in a postmenopausal osteoporosis animal model. (**A**) Animal experimental design of DLE, (**B**) uterus weight, and (**C**) osteocalcin levels in the serum. (** *p* < 0.01; **** *p* < 0.0001; ns, not significant).

To further evaluate the effects of DLE on osteoporosis, OVX mice were administered 40 and 100 mg kg^−1^ of DLE (Figure 4A). Removal of ovaries (OVX) depleted the female sex hormones, which reduced the weight of the uterus; however, E+P treatment recovered the uterus weight (Figure 4B, *p <* 0.01). DLE treatment did not alter the uterus weight. Serum OCN levels were decreased by OVX treatment but recovered by DLE treatment (Figure 4C, *p <* 0.0001).

**Figure 5:**
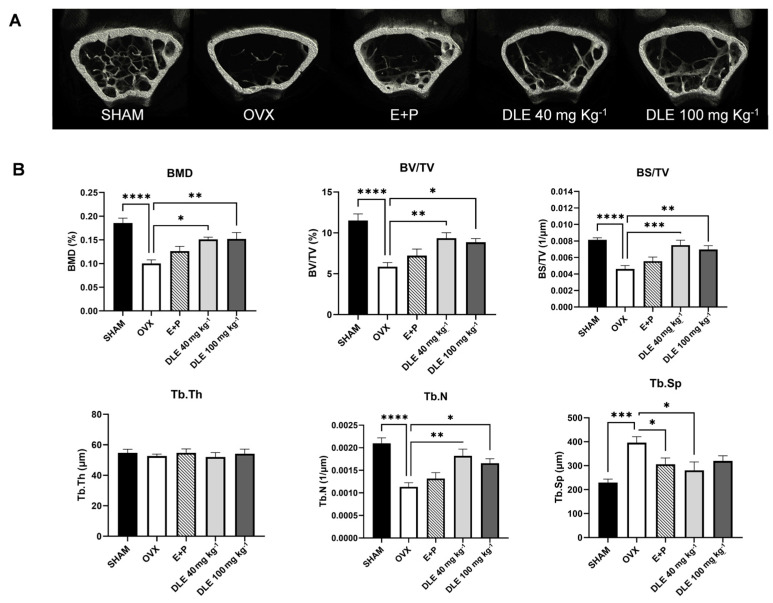
Micro-CT analysis of femurs from ovariectomized mice treated with DLE. (**A**) Micro-computed tomography images of the distal femoral region, (**B**) tomographic measurements of BMD, BV/TV, BS/TV, Tb.Th, Tb.N, and Tb.Sp (* *p* < 0.05, ** *p* < 0.01, *** *p* < 0.001; **** *p* < 0.0001).

To check the bone status, micro-CT of the femur was performed (Figure 5A). The BMD was reduced by OVX and recovered by DLE *(p <* 0.01), and the BV/TV, BS/TV, and Tb.N were decreased by OVX and increased by DLE (Figure 5B, *p <* 0.05). In contrast, Tb. Sp was increased by OVX and decreased by 40 mg kg^−1^ of DLE (Figure 5B, *p <* 0.05). Other bone markers were recovered after DLE treatment; however, the changes were not statistically significant. Changes in bone osteogenic signaling were estimated using primary cells isolated from femurs.

**Figure 6:**
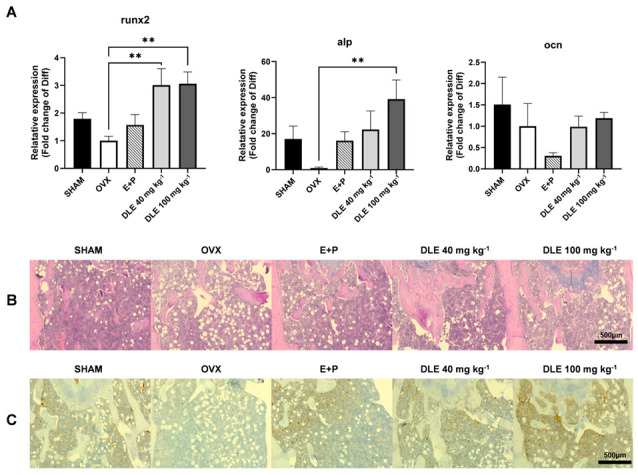
Effects of DLE on primary bone marrow cells and bone sections. (**A**) mRNA expression of osteogenic markers in primary bone marrow cells, (**B**) H&E staining and (**C**) type 1 collagen staining of the distal femoral region (** *p* < 0.01).

Both *runx2* and *alp* mRNA expression levels were significantly decreased in OVX mice but elevated by DLE (Figure 6A, *p <* 0.01). Immunohistochemical staining of bone sections revealed that DLE suppressed the bone loss and induced the expression of COL1A1 (Figure 6B,C).

Ovariectomy (OVX), the surgery removing both ovaries from female mice, is one of the representative animal models to mimic the menopause [32]. One impact of OVX is bone loss following osteoporosis. Fourteen weeks after OVX, BMD was decreased by 46%, but after DLE treatment, BMD was reduced by 18–19%. In addition, DLE-treated primary cells and immune-stained bone tissue showed osteogenic activity. These data suggest two possible modes of action of DLE: one is that DLE induces osteoblast differentiation itself and the other is that DLE induces estrogen signaling. However, an in vitro study showed that the effect of DLE was not totally compensated for with the inhibitor of estrogen signaling, and the uterus weight in the animal study indicates that DLE did not modulate ERα-mediated estrogen signaling like other phytoestrogens. Therefore, our data suggest that the effect of DLE results from an elevation in osteoblast differentiation possibly mediated by estrogen signaling. DL is known to have anti-oxidant, anti-tumorigenic and anti-diabetic properties [11,12,13,14]. Unfortunately, some common major compounds were isolated in several previous research studies depending on the solvents for extraction. Still, anti-oxidant and anti-inflammatory effects of DL were found. DL modulates inflammatory cytokines and anti-oxidant enzymes. Although these do not exhibit direct interactions with osteoblasts or bone health, this suggests further studies on the immune system and gut health are needed. Depletion in female sex hormones in the menopause impairs the gut barrier and alters the gut microbiome, which subsequently change the immune status and related cytokines [33]. This alteration influences bone metabolism via TGFβ and TNFα, which are cytokines modulating osteoblast and osteoclast fates. Therefore, previous data on the effect of DL confirmed our data on the TGFβ pathway.

While we reported DLE as a good inducer of osteoblast differentiation, there are several limitations in our study. First, we did not find all compounds in DLE. According to a chemical analysis, we found three major and some minor compounds. Due to a limitation in accessing large-scale plant samples, we could not separate all compounds, so there are no data on two major and minor compounds. Only myricitrin, which we could purchase, was tested in this study. Second, we did not assess mechanisms in detail. If we identified all compounds and traced them and their derivatives in the serum or other tissues, we could have elucidated how DLE and its components impact the development of osteoporosis. Still, we did not conduct these experiments due to sample limitations, the commercially available chemical standards and the insufficient previous research on byproducts from DLE compounds. Still, as we demonstrated, induction of osteoblast differentiation is a great choice to treat osteoporosis; our results suggest DLE, as an inducer of osteoblast differentiation, could be a novel candidate to treat osteoporosis.

## 4. Conclusions

Here, we identified DLE as a potential inducer of osteoblast differentiation and bone formation in vitro and in vivo. DLE suppressed bone loss and maintained bone density in a postmenopausal animal model, accompanied by elevated levels of bone markers involved in osteogenic signaling, partially mediated by estrogen signaling. Although the major components of DLE (myricetin, quercetin, and kaempferol derivatives) are known for their osteogenic activity, the effects of whole DLE were much better than those of the individual components. These effects of DLE originated from the induction of BMP2/4 and TGF β pathways. Therefore, our findings suggest DLE as a potential therapeutic for osteoporosis that can aid in the maintenance of healthy bones and immune statuses.

## Figures and Tables

**Table 1:** Sequences of PCR primers.

Gene	Forward (5′-3′)	Tm	Reverse (5′-3′)	Tm
runx2	TCCACAAGGACAGAGTCAGATTAC	58.4	TGGCTCAGATAGGAGGGGTA	57.4
alp	GATCATTCCCACGTTTTCAC	53.4	TGCGGGCTTGTGGGACCTGC	63.6
Ocn	AGACTCCGGCGCTACCTT	59.4	CTCGTCACAAGCAGGGTTAAG	58
Gapdh	AAG AGG GAT GCT GCC CTT AC	57.4	CCATTTTGTCTACGGGACGA	55.4

## Data Availability

The original contributions presented in the study are included in the article, further inquiries can be directed to the corresponding author.
